# CDC20 and CCNB1 Overexpression as Prognostic Markers in Bladder Cancer

**DOI:** 10.3390/diagnostics15010059

**Published:** 2024-12-29

**Authors:** Hatice Sevim Nalkiran, Ilknur Biri, Ihsan Nalkiran, Hakki Uzun, Sumeyye Durur, Recep Bedir

**Affiliations:** 1 Department of Medical Biology, Faculty of Medicine, Recep Tayyip Erdogan University, 53100 Rize, Türkiye; hatice.sevim@erdogan.edu.tr (H.S.N.); ilknurbiri.8@gmail.com (I.B.); sumeyyedurur@gmail.com (S.D.); 2 Department of Urology, Faculty of Medicine, Recep Tayyip Erdogan University, 53100 Rize, Türkiye; hakki.uzun@erdogan.edu.tr; 3 Department of Medical Pathology, Faculty of Medicine, Recep Tayyip Erdogan University, 53100 Rize, Türkiye; recep.bedir@erdogan.edu.tr

**Keywords:** bladder cancer, CDC20, Cyclin B1, tumor grade, cancer stage, overall survival

## Abstract

**Background:** Bladder cancer (BC) is one of the ten most common cancers worldwide, with a high recurrence rate and significant variation in clinical outcomes based on tumor grade and stage. This study aimed to investigate the gene expression profiles at different cancer stages to assess their potential prognostic value. **Methods:** RNA was extracted from paraffin-embedded BC tissues and the gene expression levels of CDC20 and CCNB1 were analyzed using qRT-PCR. A total of 54 BC patient samples were included in the analysis and categorized into low-grade (LG) (*n* = 23) and high-grade (HG) (*n* = 31) tumors, as well as stages pTa, pT1, and pT2. **Results:** CDC20 gene expression was significantly higher in the HG group (mean fold-change: 16.1) compared to the LG group (mean fold-change: 10.54), indicating a significant association with tumor grade (*p* = 0.039). However, no significant differences were observed in CDC20 expression across the cancer stages. For CCNB1, while gene expression was significantly elevated in higher-stage tumors (pT2 vs. pTa; *p* = 0.038), no significant association was found between CCNB1 expression and tumor grade. Survival analysis revealed that increased CCNB1 expression and advanced cancer stage were associated with poorer overall survival, whereas no significant impact of CDC20 expression or tumor grade on survival was observed. Correlation analysis indicated a positive relationship between CDC20 expression and tumor grade (r = 0.284, *p* = 0.038) and between CCNB1 expression and tumor stage (r = 0.301, *p* = 0.027). **Conclusions:** Our findings suggest that CDC20 overexpression is linked to higher tumor grades, while CCNB1 overexpression is associated with more advanced cancer stages in BC. These results underscore the potential utility of CDC20 and CCNB1 as biomarkers for tumor prognosis and as therapeutic targets. Further studies with larger cohorts are needed to validate these findings and better understand the molecular mechanisms driving BC progression.

## 1. Introduction

Bladder cancer (BC) is predominantly a uroepithelial carcinoma among the ten most common cancer types worldwide and is characterized by the formation of malignant cells in the bladder. BC also stands as the second leading cause of death among urological cancers, highlighting its substantial clinical burden [1,2,3]. BC was the fourth leading cancer in men in 2023, representing 6% of estimated new cancers and 4% of cancer-related deaths [4,5]. A spectrum of situations, ranging from recurrent chronic noninvasive tumors to advanced aggressive stages, requires multiple treatments [6]. Despite advancements in diagnosis and treatment, the disease presents significant challenges due to its high recurrence rate, variable progression, and the lack of reliable prognostic biomarkers.

Technically, uroepithelial carcinomas include tumors in the bladder, upper urinary tract (renal pelvis and ureters), and proximal urethra; however, most of them are concentrated in the bladder. Histologically, most BC cases are pure uroepithelial carcinomas, although the diagnosis of histological variants is increasing [7,8]. At diagnosis, urothelial cancer is categorized as either non-muscle-invasive BC (NMIBC; stages Tis, Ta, and T1) or muscle-invasive BC (MIBC; stages T2–T4) when the disease has grown into the muscularis propria. The overall categorization of the disease into NMIBC or MIBC is used frequently as treatment modalities differ substantially between these entities; nearly 75% of the noninvasive papillary tumors detected are pTa. The remaining 25% have an invasion of the lamina propria or pT1. A pT2 lesion is considered invasive because it disrupts the basement membrane and infiltrates the muscularis layer of the bladder wall [9]. Various tumor stages are associated with different genetic features that can be used as markers for minimally invasive diagnostics and disease aggressiveness. Treatment of BC typically involves a combined approach. The choice of treatment depends on whether the disease is muscle invasive. For NMIBC, transurethral resection of the bladder tumor is the primary treatment option, followed by intravesical immunotherapy with Bacillus Calmette-Guérin. Treatment options for MIBC include neoadjuvant therapy, radiotherapy, and radical cystectomy [10,11]. The importance of biomarkers in disease management will further increase as molecular markers become more predominant in diagnosis, treatment selection, and follow-up planning.

CDC20 and CCNB1 are cyclin proteins, and their overexpression is linked to the activation of CDC2 kinase, which has the ability to bypass p53-dependent G2/M cell cycle arrest [12]. Overexpression of the CDC20 gene disrupts the spindle assembly checkpoint and triggers premature destruction of Pds1/securin, resulting in aneuploidy [13]. Dysfunction of the CDC20-Anaphase Promoting Complex/Cyclosome (APC/C) complex frequently leads to chromosomal instability, which can contribute to the development of human diseases or increase the risk of normal cells becoming malignant [14,15]. CCNB1 is a critical regulator of CDK1 activity during mitosis, and is involved in mitotic entry, nuclear envelope breakdown, and spindle formation. CCNB1 forms a complex with CDK1 to facilitate the transition from the G2 phase to the M phase of the cell cycle [16]. The degradation of CCNB1, which leads to inactivation of CDK1 kinase activity, is essential for mitotic exit and subsequent DNA replication in the next cell cycle. The regulation of mitotic events is intricately connected to the control of the CCNB1–CDK1 complex activity, enabling cells to enter mitosis, pause at the G2 phase, or bypass mitosis under various conditions. In cancer cells, CCNB1 levels are frequently dysregulated and significantly elevated [17].

Elevated levels of CDC20 mRNA expression have been reported in human urothelial carcinoma of the bladder [18]. Overexpression of these genes has been reported to be associated with poor prognosis and increased invasive properties of cancer cells [19,20]. However, studies investigating the overexpression of CDC20 and CCNB1 have yielded varying results depending on cancer type. An increase in gene expression studies in cancer is of great importance to enable more conclusive and comprehensive research.

The aim of this study is to evaluate the relative changes in the expression levels of CDC20 and CCNB1 genes among tumor grades and cancer stages in patients with BC. Despite the existing research on the roles of CDC20 and CCNB1 in cell cycle regulation, there remains a lack of comprehensive studies specifically linking their expression levels to clinical outcomes in BC, highlighting the need for further investigation to clarify their potential as biomarkers for treatment response and early diagnosis. Specifically, we seek to understand the connection between CDC20 and CCNB1 and higher tumor grade and cancer stage. Our research aims to contribute to the development of more effective strategies in the treatment processes of BC by determining the prognostic value of these genes.

The results of this study may provide significant contributions to the treatment and early diagnosis processes of BC. Identifying the expression levels of CDC20 and CCNB1 genes can help better predict the prognosis of the disease. This, in turn, may enable the earlier detection of at-risk individuals and the development of personalized treatment approaches. Furthermore, evaluating these genes as therapeutic targets could enhance treatment strategies, thereby improving patient outcomes and quality of life.

## 2. Methods

### 2.1. Clinicopathological Features

This study received approval from the Recep Tayyip Erdogan University (RTEU) Non-Interventional Clinical Research Ethics Committee under decision number 2022/219. This study was conducted to examine the gene expression levels of CDC20 and CCNB1 using RNA samples extracted from formalin-fixed-paraffin-embedded tumor tissues of 62 patients diagnosed with BC at the RTEU Research and Training Hospital. Eight samples were excluded, leaving 54 patient samples for analysis. The exclusions were due to missing patient data, high RNA quality, and Cp expression values above 35. The clinicopathological features of the 54 patients whose CDC20 and CCNB1 gene expressions were analyzed are shown in Table 1.

### 2.2. RNA Quality Control by Agarose Gel Electrophoresis

After RNA isolation from paraffin-embedded tumor tissue samples using the Monophasic Phenol and Guanidine Isothiocyanate method, RNA samples stored in a −80 °C freezer were analyzed for quality control by running them on agarose gel electrophoresis. A 1.2% agarose gel was prepared. The mixture was poured into a gel tray, and a comb was placed to create the wells. The gel was allowed to solidify at room temperature for 30 min. After 30 min, the comb was carefully removed from the gel. The gel tray was then placed in a tank containing a 1X TAE electrophoresis system. Samples were prepared by mixing 8 µL of RNA sample with 2 µL of 6X loading buffer in a 96-well plate. A total of 10 µL of the mixture was loaded into the gel wells. The samples were run for 45 min at 70 V in 1X TAE buffer. After electrophoresis, the gel was visualized under UV light using a gel documentation system.

### 2.3. cDNA Synthesis

The cDNA synthesis was performed using the High-Capacity cDNA Reverse Transcription Kit (Applied Biosystems, Waltham, MA, USA), which utilizes random primers to bind to all RNA molecules in the sample, ensuring high-yield cDNA synthesis. RNA isolated from paraffin-embedded tissues was retrieved from −80 °C storage, and the samples were diluted to equal concentrations (1500 ng/µL) using RNase-free water from the Macherey-Nagel Nucleospin RNA Isolation Kit. For each sample, a reverse transcription mixture was prepared on ice, consisting of 1 µL 10X RT Buffer, 0.4 µL 25X dNTP mix, 1 µL 10X Random Primer, 0.5 µL MultiScribe Reverse Transcriptase, and 2.1 µL Nuclease-Free Water. A total of 5 µL of this mixture was combined with 5 µL of diluted RNA in a PCR tube, mixed by pipetting, and centrifuged for 20 s. The tubes were placed in a thermal cycler, and cDNA synthesis was initiated under the following conditions: 25 °C for 10 min, 37 °C for 120 min, and 85 °C for 5 min, followed by a 4 °C hold. The total reaction volume for each sample was 10 µL.

### 2.4. Quantitative Real-Time Polymerase Chain Reaction (qRT-PCR)

After cDNA conversion, the expression levels of CDC20 and CCNB1 genes in bladder tumor tissues were analyzed using the Light Cycler 480 II (Roche) system with the Light Cycler 480 Probes Master commercial kit. The 2X concentrated mix of the kit was optimized for a consistent MgCl_2_ concentration, which is suitable for nearly any primer combination. Template DNA, PCR primers, RNase-free water, and hydrolysis probes were added to the reaction mixture according to the following protocol: for each reaction, 0.25 µL GAPDH Forward Primer (5.0 nmol), 0.25 µL GAPDH Reverse Primer (5.0 nmol), 0.25 µL GAPDH YAK Dye-Probe (5.1 nmol), 6.0 µL Light Cycler 480 Probes Master Mix (2X), 0.5 µL TaqMan Target Primer-Probe (20X), and 1.75 µL RNase-free H_2_O were used. The total volume of the reaction mix was 9 µL, and 1 µL of cDNA (ng/µL) was added to a final reaction volume of 10 µL. Before starting the experiment, all the samples and components were thawed on ice. CDC20 (0000075822 Roche) and CCNB1 (0000075823 Roche) probes were used, with the GAPDH gene serving as the reference for normalization of mRNA expression. The wells were sealed with a Light Cycler 480 Sealing Foil, and the plate was loaded into the device to initiate the qRT-PCR run. After loading the samples into the device, the protocol Table 6 was entered into the LightCycler 480 SW 1.5.1 program. The program consisted of three stages: pre-incubation, amplification, and cooling. The pre-incubation phase was conducted at 95 °C for 10 min with a peak ramp rate of 4.4 °C/s. The amplification phase included 45 cycles, starting with denaturation at 95 °C for 10 s (ramp rate of 4.4 °C/s), followed by annealing at 60 °C for 30 s (ramp rate of 2.2 °C/s), and extension at 72 °C for 1 s (ramp rate of 4.4 °C/s). Finally, the cooling phase was performed at 40 °C for 30 s with a ramp rate of 2.2 °C/s. The results were analyzed using the “Basic Relative Quantification for All Samples” method in LightCycler 480 II software 1.5.1, and the relative expression levels were calculated using the 2^−ΔΔCt^ formula.

### 2.5. Statistical Analysis

The statistical analysis of the study data was performed using IBM SPSS Statistics Version 25. The fold changes in CDC20 and CCNB1 gene expression were calculated relative to the housekeeping gene GAPDH (ΔCt), and ΔΔCt values were derived by comparing the fold changes to non-cancerous samples (tonsils). The fold-change was determined using the 2^^(−ΔΔCt)^ method. The relative fold-change data for CDC20 and CCNB1 were examined for normal distribution using the Kolmogorov−Smirnov and Shapiro−Wilk tests. A *p*-value of >0.05 indicated a normal distribution, while a *p*-value of <0.05 indicated a non-normal distribution.

In addition to analyzing all samples together, data were also examined separately by high-grade (HG) and low-grade (LG) tumor classifications, as well as by cancer stage (pTa, pT1, and pT2), and it was determined that the data did not follow a normal distribution. Histogram plots and distribution curves were used to visualize the data distribution. Differences in gene expression fold changes between the HG and LG groups were assessed using the Mann−Whitney U test. For comparing means among two or more groups in the cancer stages pTa, pT1, and pT2, the Kruskal−Wallis test was employed. Pairwise comparisons (pTa vs. pT1, pTa vs. pT2, pT1 vs. pT2) were conducted using the Mann-Whitney U test. *p*-values of <0.05 were considered statistically significant.

Correlations between variables were evaluated using the Spearman’s correlation test. The correlation coefficient ranges from −1 to +1, where values greater than zero indicate a positive relationship between gene expression and parameter values, and values less than zero indicate a negative relationship.

## 3. Results

### 3.1. Distribution Results of Gene Expression Analysis of CDC20 and CCNB1 Genes Performed Using qRT-PCR

The expression levels of CDC20 and CCNB1 genes were analyzed using qRT-PCR with the TaqMan probe technique on a Light Cycler 480 II (Roche) device, following cDNA conversion from RNA isolated from FFPE tissues of BC patients. The expression data for the CDC20 and CCNB1 genes from each sample were normalized using the expression results of the GAPDH reference gene, and the 2^−ΔΔCt^ formula was applied to statistically evaluate the fold changes in expression. Kolmogorov-Smirnov and Shapiro-Wilk tests were performed to assess the normality of data distribution.

Of the 54 patients included in our study, 23 were in the LG group, and 31 were in the HG group. Normality tests were applied to all samples for CDC20 (Supplementary Figure S1) and separately for the LG and HG samples. The distributions were found to be *p* < 0.05, indicating that the data did not follow a normal distribution (Supplementary Figure S1). Additionally, 36 of the 54 patients had pTa, 6 had pT1, and 12 had pT2 cancer stages. When the Kolmogorov-Smirnov and Shapiro-Wilk tests were applied for CDC20, the pT1 group, which included six patient samples, showed a normal distribution (*p* > 0.05), whereas the pTa and pT2 groups did not follow a normal distribution (*p* < 0.05) (Supplementary Figure S1).

For CCNB1, normality tests were applied to all samples (Supplementary Figure S2) as well as to samples from the LG and HG groups. The distributions were found to be *p* < 0.05, indicating that the data did not follow a normal distribution (Supplementary Figure S2). When Kolmogorov-Smirnov and Shapiro-Wilk tests were applied for CCNB1 in the pTa, pT1, and pT2 cancer stage groups, the pT1 group, which included six patient samples, showed a normal distribution (*p* > 0.05), whereas the pTa and pT2 groups did not follow a normal distribution (*p* < 0.05) (Supplementary Figure S2).

### 3.2. Analysis of CDC20 and CCNB1 Gene Expression Fold-Change Results Using qRT-PCR in Tumor Grade and Cancer Stage Groups

**Figure 1:**
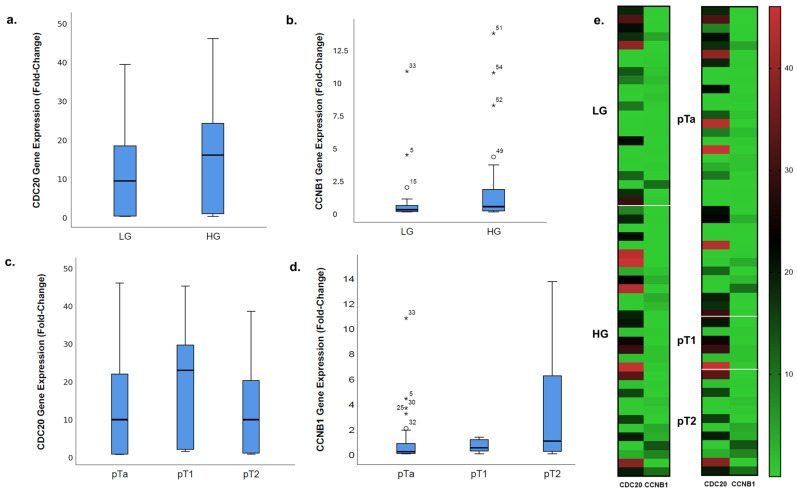
Differential expression of CDC20 and CCNB1 genes in BC tissues across different grades and stages. (**a**) CDC20 gene expression fold-change comparison between LG and HG BC tissues. (**b**) CCNB1 gene expression (fold-change) comparison between LG and HG BC tissues. (**c**) CDC20 gene expression fold-change in BC tissues across pathological stages (pTa, pT1, and pT2). (**d**) CCNB1 gene expression fold-change in BC tissues across pathological stages (pTa, pT1, and pT2). (**e**) Heatmap visualization of CDC20 and CCNB1 gene expression levels in BC tissues categorized by grade (LG and HG) and stage (pTa, pT1, and pT2). Red indicates higher expression and green indicates lower expression levels. LG: low-grade tumor, HG: high-grade tumor. The median value is indicated by a bold black line.

The fold-change in CDC20 gene expression in the LG and HG groups is illustrated in Figure 1a. The mean fold-change was found to be 10.54-fold for the LG group (95% CI for the mean, 5.28–15.81) and 16.1-fold for the HG group (95% CI for the mean, 10.30–21.91). When the average fold-change was compared between the LG (*n* = 23) and HG (*n* = 31) groups using the Mann−Whitney U test, the difference was found to be statistically significant (*p* = 0.039) (Table 2). The CDC20 gene expression was significantly higher in the HG patient group.

The fold-change in CCNB1 gene expression in the LG and HG groups is illustrated using a box plot (Figure 1b). The mean fold-change was 0.96-fold in the LG group (95% CI for the mean, −0.051 to 1.97) and 1.86-fold in the HG group (95% CI for the mean, 0.65 3.06). When the average fold-change was compared between the LG (*n* = 23) and HG (*n* = 31) groups using the Mann−Whitney U test, no statistically significant difference was found (*p* = 0.134) (Table 2).

The fold-change in CDC20 gene expression in the pTa, pT1, and pT2 groups is illustrated using a box plot (Figure 1c). The mean fold-change for CDC20 was 13.1-fold in the pTa group (95% CI for the mean, 8.18–18.03), 20.33-fold in the pT1 group (95% CI for the mean, 2.42–38.24), and 12.33-fold in the pT2 group (95% CI for the mean, 3.72–20.94). When the average fold-change was compared between the pTa (*n* = 36), pT1 (*n* = 6), and pT2 (*n* = 12) groups using the Kruskal−Wallis test, no statistically significant difference was found (*p* = 0.245) (Table 2).

The fold-change in CCNB1 gene expression for the pTa, pT1, and pT2 groups is illustrated using a box plot (Figure 1d). The mean fold-change for CCNB1 was 0.91-fold in the pTa group (95% CI for the mean, 0.24–1.59), 0.62-fold in the pT1 group (95% CI for the mean, 0.082–1.16), and 3.58-fold in the pT2 group (95% CI for the mean, 0.58–6.58). When the average fold-change was compared between the pTa (*n* = 36), pT1 (*n* = 6), and pT2 (*n* = 12) groups using the Kruskal−Wallis test, no statistically significant difference was found (*p* = 0.088) (Table 2). No significant difference was found between the fold changes in CCNB1 gene expression among the pTa, pT1, and pT2 groups. Therefore, Dunn’s test, which is used to assess post-hoc differences within groups following the Kruskal−Wallis test, could not be applied. However, since the *p*-value of 0.088 approached statistical significance, comparisons between subgroups were conducted using the Mann−Whitney U test. The results were as follows: pTa-pT1, *p* = 0.332 (Table 2); pTa-pT2, *p* = 0.038 (Table 2); and pT1-pT2, *p* = 0.385 (Table 2). A statistically significant fold-change in CCNB1 gene expression was found between the pTa and pT2 patient groups. Increased CCNB1 expression was also observed in the pT2 group.

Heatmap analysis (Figure 1e) revealed consistent upregulation of CDC20 and CCNB1 genes in HG and higher-stage BC tissues. High expression levels (marked in red) were more prevalent in HG and advanced stages (T1 and T2), while LG and early-stage (pTa) samples showed predominantly lower expression levels (green).

In summary, the fold increase in CDC20 gene expression was found to be significantly higher in the patient group with a high tumor grade. No significant difference was observed in the fold-change in CDC20 gene expression among cancer stage groups. The fold increase in CCNB1 gene expression did not show a significant difference between the tumor grade groups. However, a statistically significant difference was observed between the pTa and pT2 cancer stage groups, with the pT2 group showing a higher fold increase in CCNB1 gene expression.

### 3.3. Correlation

Based on the data presented in the correlation table, Spearman’s rho correlation analysis was conducted to assess the relationships between the expression of CDC20 and CCNB1 genes and tumor characteristics such as tumor stage (pTa, T1 andT2), grade (LG and HG), and overall survival (months). The results showed that CDC20 expression had a significant positive correlation with tumor grade (r = 0.284, *p* = 0.038), indicating that higher CDC20 expression was associated with higher tumor grades (Table 3). However, no significant correlation was observed between CDC20 expression and tumor stage (pTa, T1 and T2) (r = 0.127, *p* = 0.359) or overall survival (r = −0.069, *p* = 0.622).

CCNB1 expression, on the other hand, exhibited a significant positive correlation with tumor stage (r = 0.301, *p* = 0.027), suggesting that higher CCNB1 expression was related to more advanced tumor stages. Moreover, CCNB1 expression exhibited a negative correlation with overall survival (r = −0.259, *p* = 0.059), indicating that increased CCNB1 expression is associated with shorter survival times, though the correlation did not reach statistical significance. No significant correlation was found between CCNB1 expression and tumor grade (r = 0.206, *p* = 0.136). Furthermore, a strong positive correlation was observed between tumor stage and tumor grade (r = 0.598, *p* < 0.001), highlighting that more advanced tumor stages were associated with higher tumor grades. Tumor grade also showed a significant negative correlation with overall survival (r = −0.275, *p* = 0.044), indicating poorer survival outcomes with higher tumor grades. Similarly, tumor stage exhibited a significant negative correlation with overall survival (r = −0.407, *p* = 0.002), suggesting that advanced tumor stages are associated with shorter survival times.

These findings suggest that, while CDC20 expression is more closely related to tumor grade, CCNB1 expression may play a greater role in tumor stage progression. Both tumor grade and stage significantly impact overall survival in patients with BC. 

### 3.4. Survival Data Subgroups

Survival data were used to compare various patient subgroups, including tumor stage (pTa, pT1, and pT2), grade (LG and HG), and gene expression levels of CDC20 and CCNB1, with respect to overall survival (Table 4). In the pTa group (*n* = 36), 28 events occurred and the median survival was 64.65 months, with a mean survival of 62.85 months (95% CI: 56.77–68.94). For pT1 patients (*n* = 6), four events were recorded, with a median survival of 60.75 months, and a mean of 55.08 months (95% CI: 39.73–70.43). The pT2 group (*n* = 12) showed a mean survival of 32.25 months (95% CI: 12.23–52.27) and a median survival of 14.25 months, with four events. Patients with LG (*n* = 23) had a mean survival of 64.44 months (95% CI: 57.45–71.43), while those with HG (*n* = 31) had a shorter mean survival of 48.32 months (95% CI: 38.27–58.38), with a median of 60.40 months.

For CDC20, patients with downregulated expression (*n* = 27) had a mean survival of 57.37 months (95% CI: 47.92–66.81) and a median survival of 63.70 months. Those with upregulated CDC20 (*n* = 27) had a mean survival of 53.01 months (95% CI: 43.00–63.02) and a median of 62.80 months. In the case of CCNB1, patients with downregulated expression (*n* = 27) had a mean survival of 59.54 months (95% CI: 49.75–69.34) and a median of 64.90 months. Upregulated CCNB1 patients (*n* = 27) had a mean survival of 50.84 months (95% CI: 41.41–60.27) and a median of 60.40 months. These findings suggest that tumor stage, grade, and gene expression levels of CDC20 and CCNB1 impact survival outcomes in these patient cohorts.

**Figure 2:**
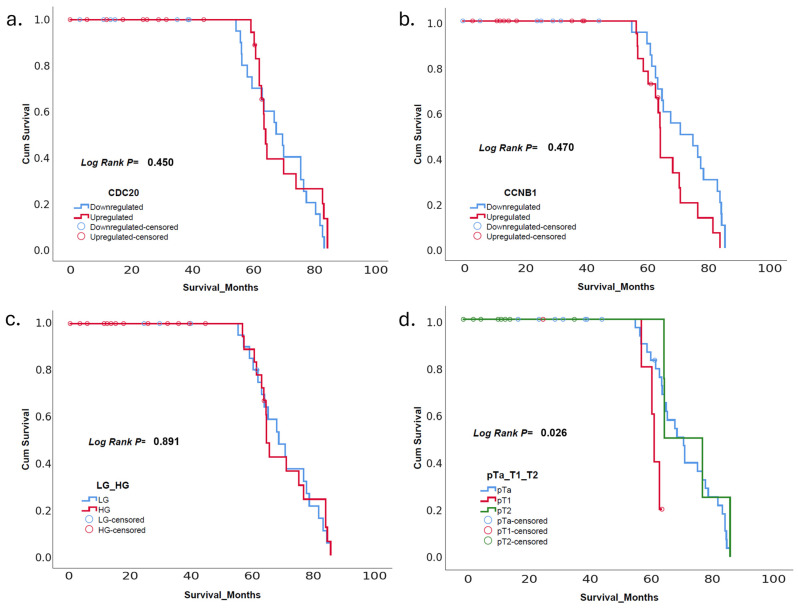
Kaplan−Meier survival curves for tumor grades and cancer stages (**a**) CDC20 expression: Survival comparison between patients with downregulated and upregulated CDC20 expression, showing no significant difference (log-rank *p* = 0.450). (**b**) CCNB1 expression: Patients with upregulated CCNB1 expression exhibit significantly worse overall survival compared to those with downregulated expression (log-rank *p* = 0.047). (**c**) Tumor grade (LG vs. HG): No significant difference in survival is observed between LG and HG tumors (log-rank *p* = 0.891). (**d**) Tumor stage (pTa, pT1, and pT2): Significant survival differences are evident across tumor stages, with more advanced stages (pT2) associated with poorer survival outcomes (log-rank *p* = 0.026).

The Kaplan−Meier survival analysis presented in Figure 2 highlights the relationship between survival and various clinical and molecular factors. Figure 2a shows the survival curves of patients with downregulated and upregulated CDC20 expression, with no significant differences observed between the groups (log-rank *p* = 0.450). In contrast, Figure 2b illustrates a statistically significant difference in survival based on CCNB1 expression, where patients with upregulated CCNB1 had worse overall survival compared to those with downregulated expression (log-rank *p* = 0.047). Figure 2c compares LG and HG tumors, revealing no significant difference in survival between the two groups (log-rank *p* = 0.891). Finally, Figure 2d demonstrates a significant survival difference across tumor stages (pTa, pT1, and pT2), with advanced stages showing poorer survival outcomes (log-rank *p* = 0.026). These results suggest that CCNB1 expression and tumor stage are important prognostic factors in this cohort, while CDC20 expression and tumor grade did not significantly impact survival.

In conclusion, our survival analysis reveals that tumor stage and molecular markers such as CCNB1 expression are critical prognostic factors in this cohort of patients. The Kaplan−Meier analysis demonstrates that the advanced tumor stage (pT2) is associated with significantly worse survival outcomes, as shown by the log-rank test (*p* = 0.026). This is consistent with the median survival times observed in the survival table, where patients with pT2 stage had the shortest median survival (14.25 months). Additionally, CCNB1 upregulation correlates with poorer survival, as reflected both in the survival curves (log-rank *p* = 0.047) and in the table, where upregulated CCNB1 is associated with a lower mean survival time (50.84 months) compared to downregulated expression (59.54 months). However, CDC20 expression and tumor grade (LG vs. HG) did not appear to significantly influence overall survival, as shown by the non-significant log-rank *p*-values (*p* = 0.450 and *p* = 0.891, respectively) and comparable median survival times in these groups. These findings suggest that while CCNB1 expression and tumor stage are important factors for predicting patient outcomes, CDC20 expression and tumor grade may be less influential in this context.

## 4. Discussion

BC is among the ten most common cancers worldwide. The majority of BC cases are of the urothelial carcinoma subtype, with other subtypes, including squamous cell carcinoma, sarcoma, lymphoma, and adenocarcinoma. Approximately 75% of BC cases are classified as NMIBC, which has a high rate of recurrence. Globally, BC occurs more frequently in men than in women, and one contributing factor to its higher incidence in men is related to occupational exposure. BC treatment typically involves combination therapy, which includes multiple treatment modalities [21].

The replication capacity of cells is crucial for the life and development of complex organisms [22]. It is known that important cytological events during mitosis drive cell cycle transitions. Many proteins are involved in facilitating these cytological events during mitosis. One such protein complex is APC/C, a large multimeric complex responsible for initiating chromatid separation and the onset of anaphase [23]. CDC20 interacts with various proteins during the cell cycle to regulate the structure of APC/C. Additionally, CDC20 plays a critical role in the regulation of immune cell infiltration and apoptosis. Alterations in CDC20 functionality have been linked to genomic instability [24,25,26]. CDC20 enhances the proliferation, invasion, and metastasis of cancer cells through the CDC20-mediated angiogenesis pathway, highlighting its role in promoting angiogenesis in cancer tissues [27].

In this study, patients diagnosed with BC were grouped according to tumor grade and cancer stage. Gene expression levels of CDC20, which has a close functional relationship with APC/C, and CCNB1, a downstream marker of the APC/C pathway, were also examined in the groups.

In the present study, 23 patients were included in the low tumor grade group and 31 patients were in the high tumor grade group. Normality tests were applied to the LG and HG samples for CDC20 expression, and the distribution test result was *p* < 0.05, indicating that CDC20 expression did not follow a normal distribution. The fold-change results for CDC20 gene expression showed an average 10.54-fold increase in the low tumor grade group and a 16.1-fold increase in the high tumor grade group. A comparison of fold-change averages using the Mann−Whitney U test revealed a statistically significant difference between the low and high tumor grade patient groups. CDC20 gene expression was significantly higher in the high tumor grade patient group than in the low tumor grade group. An association between CDC20 overexpression and tumor grade has been reported in epithelial ovarian cancer [28]. Another study has reported that CDC20 is frequently overexpressed in HG tumors in breast cancer and other HG tumor types [29]. A study investigating the relationship between CDC20 gene expression and tumor grade and stage in various cancers found a significant association between high CDC20 expression and advanced stages in breast, colon, endometrial, and prostate cancers [30]. They suggested CDC20 as a potential biomarker for tumor prognosis and a therapeutic target. Our results, which demonstrate that CDC20 overexpression is linked to higher tumor grades in BC, support these findings. Overexpression of CDC20 and CCNB1 has been identified in tumor tissues from breast cancer, glioblastoma, ovarian cancer, and ductal adenocarcinoma. In addition to its role in mitotic processes, CDC20 overexpression was found to enhance the invasive properties of glioblastoma stem cell-like cells [31]. CDC20 overexpression may play a crucial role in the development and progression of pancreatic ductal adenocarcinoma, suggesting that CDC20 could serve as a prognostic marker and a potential therapeutic target [32]. Furthermore, CDC20 overexpression has been associated with poor prognosis in breast and ovarian cancers [19,20]. In a systematic analysis of various genes using cDNA microarray, the CDC20 gene was included. Quantitative real-time PCR analysis of nine BC tissues showed that eight of them exhibited more than a fivefold increase in CDC20 expression compared to normal tissues [18].

Of the 54 patients included in this study, 36 had pTa, 6 had pT1, and 12 had pT2 cancer stages. No significant relationship was found between high CDC20 overexpression and high cancer stage. Consistent with our findings, a study involving 332 samples, including squamous cell carcinoma of the uterus and squamous intraepithelial lesions, found no significant correlation between CDC20 overexpression and advanced cancer stage [33]. Another study on oral squamous cell carcinoma found no significant relationship between CDC20 overexpression and advanced cancer stage [34]. Conversely, a study of 131 FFPE gastric cancer tissues identified a positive correlation between CDC20 overexpression and advanced cancer stage [35].

CDC20 is often overexpressed in multiple cancer types and exhibits oncogenic characteristics [36,37]. The overexpression of CDC20 in NSCLC indicates its potential as both a prognostic and predictive marker for the disease [38]. Elevated CDC20 expression is strongly associated with visceral pleural invasion and reduced survival rates in male patients with NSCLC [38]. Supporting this idea, increased CDC20 expression has been observed in lung adenocarcinoma tissues compared to normal lung tissue samples [39]. Notably, silencing of CDC20 caused cell cycle arrest at the G2/M phase and diminished the colony-forming ability of lung cancer cells [18]. Additionally, CDC20 plays a crucial role in breast cancer progression, with high expression levels in a variety of breast cancer cell lines and HG primary breast carcinoma tissues [40]. Similarly, an independent study demonstrated elevated CDC20 expression in breast cancer patients by analyzing 445 cases with 20 years of follow-up data [19]. In addition, silencing CDC20 in pancreatic carcinoma cells with siRNA inhibited cell proliferation and triggered G2/M phase arrest [41].

CDC20 has been implicated in colorectal cancer, and its expression levels are positively correlated with advanced clinical stages, metastasis, and decreased survival rates, highlighting its potential as a key biomarker for diagnosing and predicting the prognosis of the disease [42]. Furthermore, another study revealed that CDC20 expression increased more than fivefold in 77% of colorectal cancer tissues [18]. However, a separate independent study reported that CDC20 expression was lower in colorectal cancer tissues compared to normal tissues [43].

Li et al. reported that CDC20 was overexpressed in 68% of hepatocellular carcinoma cases compared to that in adjacent normal tissues. Notably, its expression is positively associated with sex, tumor differentiation, and tumor-node-metastasis (TNM) stage [44]. CDC20 is overexpressed in various gastric cancer tumor tissues [45]. In gastric cancers, CDC20 upregulation is positively associated with tumor size, histological grade, and TNM stage, while its elevated expression is also correlated with worse survival outcomes [35]. Glioblastoma, an aggressive form of brain cancer, shows elevated CDC20 expression, whereas its levels are reduced in LG tumors [46]. Similarly, in BC patients, CDC20 expression is positively associated with age, advanced tumor stage, HG tumor, distant metastasis, shorter recurrence-free survival, and worse overall survival [47]. Additionally, Mondal et al. reported elevated CDC20 expression in several oral squamous cell carcinoma cell lines and primary head and neck tumors [48,49]. Elevated CDC20 expression in oral squamous cell carcinoma cells causes premature anaphase due to disrupted APC activity, leading to genomic instability, including aneuploidy [48]. Recent reports have indicated that suppressing CDC20 in melanoma cells leads to G2/M phase arrest and inhibits cell growth [50]. The expression profiles of CCNB1 reported in the aforementioned studies are summarized in Table 5.

The cell cycle is regulated by a conserved family of cyclin-dependent kinases (CDKs) and their regulatory subunits, namely cyclins. Among these cyclins, CCNB1 is crucial as a regulatory subunit of CDK1, which is essential for the transition from G2 phase to mitosis. It has been reported that cancer cells show increased expression levels of CCNB1 [51,52,53,54,55,56]. In the present study, the analysis did not reveal a statistically significant difference in CCNB1 gene expression fold-change between the different tumor grade groups. In contrast to our findings, a study of 40 pancreatic cancer tissue samples reported a significant difference in CCNB1 expression between cancerous and normal pancreatic tissues, establishing a link between high CCNB1 expression and increased tumor grade [57]. Another study investigating the role of CCNB1 in BC across different tumor grades found that CCNB1 gene expression varied between tumor grade groups and could play a role in tumor aggressiveness [58]. While some studies have demonstrated an association between CCNB1 gene expression and high tumor grade, our findings did not show this correlation, which may be due to the limited sample size and variability in tumor characteristics within the studied patient populations.

In this study, CCNB1 gene expression analyses were performed in groups classified as LG and HG tumors. Normality tests were performed for the data obtained from the samples in each group. The results indicated that the pTa and pT2 groups did not follow a normal distribution (*p* < 0.05), while the pT1 group (*p* > 0.05) showed a normal distribution. The limited number of patients (*n* = 6) in the pT1 group necessitated the use of non-parametric statistical analysis for the pT1 group. CCNB1 gene expression was found to be significantly increased in the high-stage cancer patient group. CCNB1 is a key molecule in the G2-M phase transition during the cell cycle and is overexpressed in various tumor types. Consistent with our results, a study examining tumor samples from 41 patients with stage II-IV squamous cell carcinoma of the tongue found CCNB1 overexpression in 37% of the cases [59]. A biomarker study involving 165 BC patient samples analyzed the expression levels of four genes (CCNB1, KIF4A, TPX2, and TRIP13) and their association with clinical characteristics, revealing a correlation between elevated CCNB1 expression and cancer stage [60]. However, studies have not identified a significant association between CCNB1 protein expression and TNM staging in pancreatic cancer [57].

Elevated CCNB1 expression is associated with poor prognosis in patients with squamous cell carcinomas of the esophagus [61], larynx [62], lung [63], and tongue [59]. Moreover, overexpression of CCNB1 is implicated in metastasis, likely by promoting epithelial-mesenchymal transition in colorectal tumors [64] and esophageal squamous cell carcinoma cells [61,65]. CCNB1 overexpression has been reported in a variety of human tumors, including breast cancer, cervical cancer, gastric cancer, colorectal cancer, head and neck squamous cell carcinoma, and NSCLC [63,66,67,68,69,70,71], and its upregulation is closely associated with poor prognosis in various types of cancer, including breast cancer [63,72,73].

Furthermore, CCNB1 overexpression contributes to resistance to radiotherapy in head and neck squamous cell carcinoma [71], and nuclear cyclin B1-positive breast carcinomas are resistant to adjuvant therapy [73].

Highly expressed CCNB1, even during the G1 phase, binds to its partner CDK1, which phosphorylates a range of substrates independently of the cell cycle phase, thereby promoting aggressive proliferation in neoplastic tissues [74]. Additionally, CCNB1 overexpression is associated with aneuploidy and increased proliferation in human mammary carcinomas [75]. In summary, the deregulation of CCNB1 plays a key role in neoplastic transformation and drives the proliferation of tumor cells. Conversely, downregulation of CCNB1, which reduces CDK1/CCNB1 activity, could inhibit the aggressive proliferation of tumor cells. Targeting CCNB1 function inhibits the proliferation of human tumor cells [76,77]. The expression profiles of CCNB1 reported in the aforementioned studies are summarized in Table 6.

CDC20 expression showed a significant positive correlation with tumor grade, indicating that higher CDC20 levels were associated with more aggressive tumor phenotypes. This aligns with previous studies that have reported the involvement of CDC20 in tumorigenesis through its role in cell cycle regulation and mitotic progression. The absence of a significant correlation between CDC20 expression and tumor stage suggests that its impact may be more closely tied to histological differentiation rather than the extent of tumor invasion or spread. Furthermore, the lack of association with overall survival highlights the possibility that the prognostic relevance of CDC20 may be limited to certain subsets of patients with BC or specific tumor characteristics.

CCNB1 expression demonstrated a significant positive correlation with tumor stage, suggesting its role in promoting tumor invasiveness and progression. As a key regulator of the G2/M transition in the cell cycle, elevated CCNB1 expression likely reflects increased proliferative activity in advanced tumor stages. Importantly, the observed negative correlation between CCNB1 expression and overall survival, although not statistically significant, underscores its potential as a prognostic marker. This finding is consistent with reports in other malignancies where high CCNB1 expression has been linked to poor clinical outcomes. However, the lack of a significant correlation with tumor grade indicates that CCNB1 may be more critical in the context of tumor invasion rather than differentiation.

The significant positive correlation between tumor stage and tumor grade reinforces the established association between higher tumor grades and more advanced stages of BC. The significant negative correlations of both tumor stage and tumor grade with overall survival further highlight their critical roles in determining patient prognosis. These findings underscore the importance of integrating molecular markers, such as CDC20 and CCNB1, with traditional pathological features to enhance prognostic accuracy.

Taken together, these results suggest distinct but complementary roles of CDC20 and CCNB1 in BC progression. While CDC20 appears to be more closely associated with tumor grade, CCNB1 is more strongly associated with tumor stage and survival outcomes. These differences highlight the potential utility of these genes as biomarkers in different aspects of tumor biology.

## 5. Clinical Implications, Future Perspectives and Conclusions

Given the involvement of CDC20 and CCNB1 in tumor progression, these proteins represent potential targets for novel therapeutic strategies. Future studies should explore the development of specific inhibitors or small molecules that can modulate the activity of CDC20 and CCNB1 either alone or in combination with existing therapies. Clinical trials assessing the safety and efficacy of such targeted therapies are crucial for advancing treatment options for patients with BC. Proteasome inhibitors interfere with the degradation of various substrates, including those reliant on APC-dependent proteolysis, thereby impacting multiple cellular processes and potentially leading to a range of side effects. Therefore, identifying and developing more selective inhibitors that specifically target CDC20 could offer an effective anticancer therapeutic strategy to address this challenge. Considering the pro-tumorigenic role of CDC20 in various human cancers, its targeting could disrupt mitosis and subsequently suppress cancer cell proliferation [78]. Several studies have used Tosyl-L-arginine methyl ester (TAME) and its cell-permeable form ProTAME, which is a small-molecule inhibitor of APC that mimics the isoleucine-arginine (IR) motif of co-activators, and functions as a competitive inhibitor to facilitate the dissociation of CDC20 from the APC core complex [79,80]. As described earlier, CCNB1 levels were decreased in cells undergoing senescence upon treatment with Adriamycin. Anticancer drug-induced senescence is one of the outcomes of cancer chemotherapy due to its inability to undergo apoptosis in many types of cancer. Thus, it is possible to evaluate clinical outcomes by measuring CCNB1 levels after treatment with anticancer drugs because senescence-associated growth arrest of cancer cells is generally considered irreversible [81].

Recent studies have revealed that the expression level of CDC20 can influence the regulation of the antitumor immune response. CDC20 has been linked to the tumor mutation burden, immune checkpoint molecules, tumor microenvironment, and immunological infiltration [82]. For instance, the A20/TNFAIP3-CDC20-CASP1 axis, which involves inflammation-related genes identified in triple-negative breast cancer, is linked to cytokine levels [83]. CDC20 plays a critical role in the antitumor immune system, such as the expression of cytokines, activation of immune cells, and expression of immune checkpoint molecules [84]. The mechanisms accounting for overexpressed CCNB1 are not yet completely understood. It has been reported that the tumor suppressors p53 and BRCA1 negatively regulate the promoter of CCNB1 [85,86,87,88], whereas the oncogene c-Myc positively regulates the expression of CCNB1 in cooperation with the loss of p53 [89]. More recently, it has been reported that both antibodies and T cells are generated in response to aberrant CCNB1 expression in tumors like breast cancer [90,91], indicating that overexpressed CCNB1 could serve as one of the signals to initiate the communication between cancer cells and their microenvironment [92].

The integration of molecular markers like CCNB1, with traditional pathological parameters, such as tumor grade and stage, could enhance the predictive accuracy of current prognostic models and support the development of personalized therapeutic strategies. Artificial intelligence (AI) can be particularly useful in analyzing liquid biopsy data, such as urine or blood samples, to detect changes in the expression of CDC20 and CCNB1. AI-based systems can identify key molecular signatures associated with BC, enabling noninvasive diagnostic methods for early detection and monitoring [93]. By analyzing the expression levels of CDC20 and CCNB1 along with other clinical and molecular factors, AI can help stratify patients based on their risk of recurrence or progression. Elevated expression of CDC20 and CCNB1 has been associated with advanced tumor stages and poor prognosis, and AI models can integrate these data to predict patient outcomes, personalize treatment plans, and identify high-risk patients who may benefit from aggressive interventions.

In conclusion, this study offers valuable insights into the gene expression profiles of CDC20 and CCNB1 in BC, uncovering significant associations between these genes and tumor characteristics. These results indicate that CDC20 overexpression is significantly associated with higher tumor grades, suggesting its potential utility as a prognostic biomarker and therapeutic target for BC. However, no significant relationship was identified between CDC20 overexpression and cancer stage, reflecting the intricate nature of gene expression regulation across different tumor microenvironments. Although CCNB1 overexpression demonstrated an association with more advanced cancer stages, it did not exhibit a statistically significant difference across tumor grade groups, likely due to limitations in sample size and tumor heterogeneity. These findings are in alignment with some existing literature, while diverging from others, emphasizing the need for further investigation to clarify the roles of CDC20 and CCNB1 in cancer progression. To validate these observations and gain a more comprehensive understanding of the molecular mechanisms driving BC, future studies should incorporate larger cohorts and a more diverse patient population.

## Figures and Tables

**Table 1:** Clinicopathological features of patients.

Features	Patient Number (*n* = 54)
Age	
Mean (minimum and maximum)	64 (29–85)
Median	66
Sex	
Female	3
Male	51
Tumor Grade	
Low-grade (LG)	23
High-grade (HG)	31
Pathological Stage	
pTa	36
pT1	6
pT2	12

**Table 2:** Analysis of CDC20 and CCNB1 gene expression across tumor grades and cancer stages.

Genes	Patient Groups	*n*	Significance	
CDC20	LG	23	0.039	Mann−Whitney U
	HG	31		
	pTa	36	0.245	Kruskal−Wallis
	pT1	6		
	pT2	12		
	pTa-pT1	36-6	0.114	Mann−Whitney U
	pT1-pT2	36-12	0.634	Mann−Whitney U
	pTa-pT2	6-12	0.160	Mann−Whitney U
CCNB1	LG	23	0.134	Mann−Whitney U
	HG	31		
	pTa	36	0.088	Kruskal−Wallis
	pT1	6		
	pT2	12		
	pTa-pT1	36-6	0.322	Mann−Whitney U
	pT1-pT2	36-12	0.349	Mann−Whitney U
	pTa-pT2	6-12	0.038	Mann−Whitney U

**Table 3:** Correlation coefficients between CDC20, CCNB1 expression, tumor stage, and tumor grade in BC.

		CDC20	CCNB1	pTa_T1_T2	LG_HG	Survival (Months)
CDC20	Correlation Coefficient	1.000	−0.241	0.127	0.284 *	−0.069
	Significance		0.079	0.359	0.038	0.622
CCNB1	Correlation Coefficient	−0.241	1.000	0.301 *	0.206	−0.259
	Significance	0.079		0.027	0.136	0.059
pTa_T1_T2	Correlation Coefficient	0.127	0.301 *	1.000	0.598 **	−0.407
	Significance	0.359	0.027		0.000	0.002
LG_HG	Correlation Coefficient	0.284 *	0.206	0.598 **	1.000	−0.275
	Significance	0.038	0.136	0.000		0.044
Overall Survival (Months)	Correlation Coefficient	−0.069	−0.259	−0.407	−0.275	1.000
	Significance	0.622	0.059	0.002	0.044	

LG: Low Grade, HG: High Grade, *p* < 0.05 is indicated with a single asterisk (*), *p* < 0.01 is indicated with a double asterisk (**)

**Table 4:** Survival data analysis of BC patients based on tumor stage, grade, and expression levels of CDC20 and CCNB1 genes.

		Number of Patients	Number of Events	Censored	Percent	Overall SurvivalMean (Months)	95% CI for Mean (Lower Bound)	95% CI for Mean (Upper Bound)	Std. Error	Overall SurvivalMedian (Months)
	pTa	36	28	8	22.2	62.85	56.77	68.94	2.99	64.65
	pT1	6	4	2	33.3	55.08	39.73	70.43	5.97	60.75
	pT2	12	4	8	66.7	32.25	12.23	52.27	9.09	14.25
	LG	23	19	4	17.4	64.44	57.45	71.43	3.37	64.9
	HG	31	17	14	45.2	48.32	38.27	58.38	4.92	60.40
CDC20 gene (Cut-off median: 10.64)	Downregulated	27	20	7	25,9	57.37	47.92	66.81	4.59	63.70
	Upregulated	27	16	11	40,7	53.01	43.00	63.02	4.86	62.80
CCNB1 gene(Cut-off median: 0.285	Downregulated	27	20	7	25.9	59.54	49.75	69.34	4.76	64.90
	Upregulated	27	16	11	40.7	50.84	41.41	60.27	4.58	60.40

**Table 5:** Overview of CDC20 Regulation in Various Cancer Types.

Cancer Type	Regulation	References
Glioblastoma	Upregulated	[27,46]
Non-small cell lung cancer (NSCLC)	Upregulated	[38,39,40]
Breast cancer	Upregulated	[18,19]
Colorectal cancer	Upregulated	[18,42]
Colorectal cancer	Downregulated	[43]
Hepatocellular carcinoma	Upregulated	[44]
Gastric cancer	Upregulated	[35,45]
Bladder cancer	Upregulated	[47]
Head and Neck Squamous Cell Carcinoma (HNSCC)	Upregulated	[48,49]

**Table 6:** Overview of CCNB1 regulation in various cancer types.

Cancer Type	Regulation	References
Esophageal squamous cell carcinoma	Upregulated	[61,65]
Larynx carcinoma	Upregulated	[62]
Non-small cell lung cancer (NSCLC)	Upregulated	[63]
Tongue carcinoma	Upregulated	[59]
Colorectal cancer	Upregulated	[64,66]
Breast cancer	Upregulated	[68,71,73]
Cervical cancer	Upregulated	[67]
Gastric cancer	Upregulated	[56]
Head and neck squamous cell carcinoma (HNSCC)	Upregulated	[70]
Mammary carcinoma	Upregulated	[75]
Lymphoma	Upregulated	[69]

## Data Availability

The data presented in this study are available on request from the corresponding author due to privacy and ethical restrictions.

## Supplementary Materials

The following supporting information can be downloaded at https://www.mdpi.com/article/10.3390/diagnostics15010059/s1, Figure S1: Normality test for all samples in CDC20; Figure S2: Distribution and normality analysis of CDC20 gene expression across LG, HG, and tumor staging groups (pTa, pT1, pT2); Figure S3: Normality test for all samples in CCNB1; Figure S4: Distribution and normality analysis of CCNB1 gene expression across LG, HG, and tumor staging groups (pTa, pT1, pT2).
