# Supplementary material for: CDC20 and CCNB1 Overexpression as Prognostic Markers in Bladder Cancer
Source: Diagnostics (Basel). 2024 Dec 29;15(1):59. doi: 10.3390/diagnostics15010059 (PMC11719780; doi:10.3390/diagnostics15010059)
Supplement: Supplementary file 1 [file diagnostics-15-00059-s001.zip › diagnostics-3293917-supplementary.pdf]

Supplementary Figures and Legends

a.

Tests of Normality

	Kolmogorov-Smirnov[a]			Shapiro-Wilk		
	Statistic	df	Sig.	Statistic	df	Sig.
CDC20	,210	54	,000	,848	54	,000

a. Lilliefors Significance Correction

b.

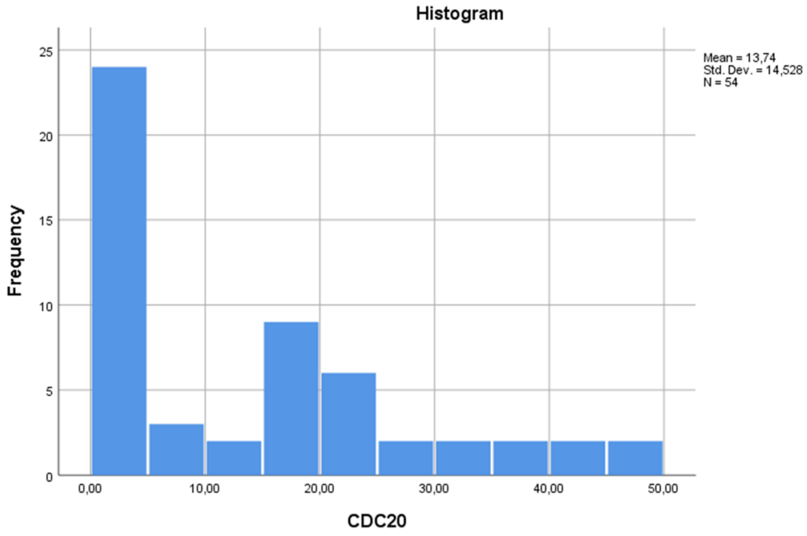

Supplementary Figure S1. Normality test for all samples in CDC20.

a.

Tests of Normality

	LG_HG	Kolmogorov-Smirnov[a] Statistic	df	Sig.	Shapiro-Wilk Statistic	df	Sig.
CDC20	LG	,278	23	,000	,828	23	,001
	HG	,208	31	,001	,859	31	,001

a. Lilliefors Significance Correction

b.

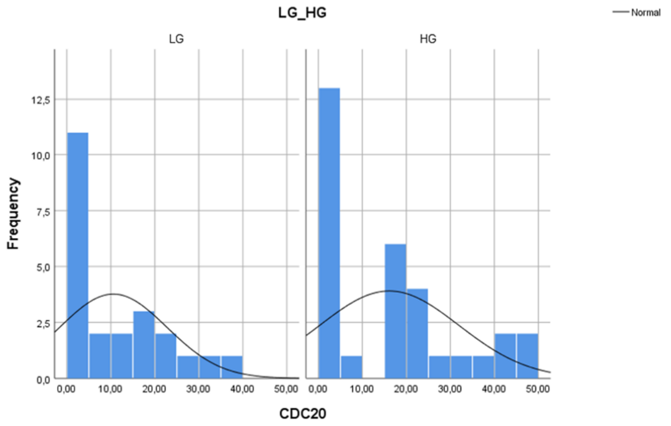

c.

Tests of Normality

	pTa_T1_T2	Kolmogorov-Smirnov[a] Statistic	df	Sig.	Shapiro-Wilk Statistic	df	Sig.
CDC20	pTa	,225	36	,000	,834	36	,000
	pT1	,200	6	,200*	,926	6	,549
	pT2	,246	12	,043	,840	12	,027

*. This is a lower bound of the true significance.

a. Lilliefors Significance Correction

d.

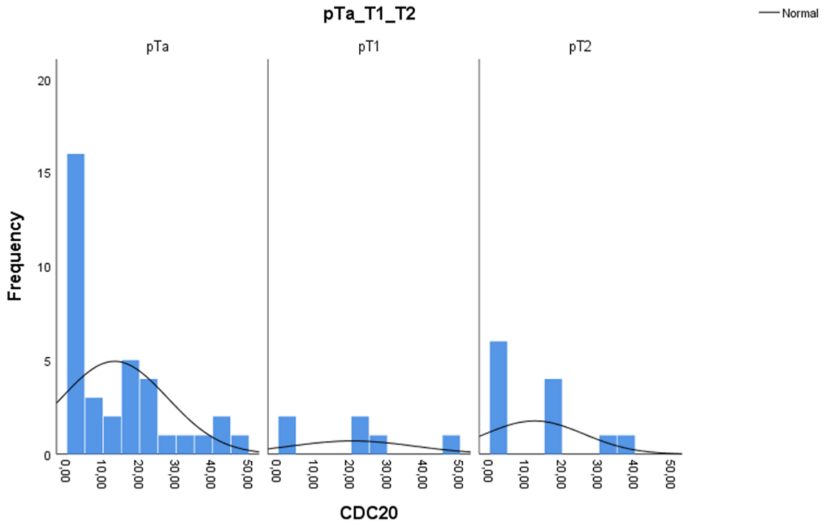

Supplementary Figure S2. Distribution and normality analysis of CDC20 gene expression across LG, HG, and tumor staging groups (pTa, pT1, pT2). **a.** Distribution of fold changes in CDC20 gene expression, **b.** Normality test for CDC20 data in LG and HG groups, **c.** Distribution of CDC20 gene expression fold-change in LG and HG groups, **d.** Normality test for CDC20 data in the pTa, pT1, and pT2 groups, **e.** Distribution of CDC20 gene expression fold-change in the pTa, pT1, and pT2 groups.

a.

Tests of Normality

	Kolmogorov-Smirnov[a]			Shapiro-Wilk		
	Statistic	df	Sig.	Statistic	df	Sig.
CCNB1	,308	54	,000	,545	54	,000

a. Lilliefors Significance Correction

b.

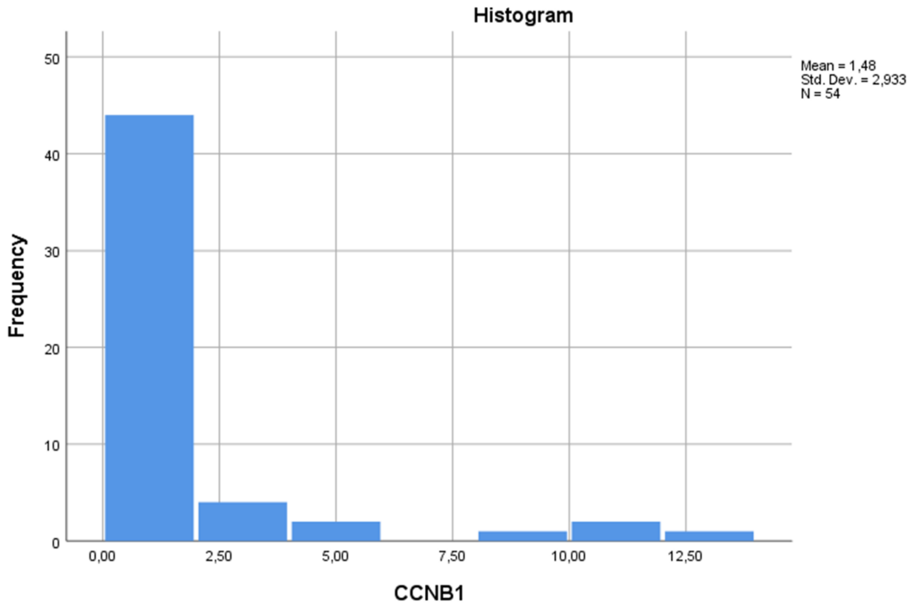

Supplementary Figure S3. Normality test for all samples in CCNB1.

a.

Tests of Normality

	LG_HG	Kolmogorov-Smirnov[a] Statistic	df	Sig.	Shapiro-Wilk Statistic	df	Sig.
CCNB1	LG	,360	23	,000	,441	23	,000
	HG	,294	31	,000	,610	31	,000

a. Lilliefors Significance Correction

b.

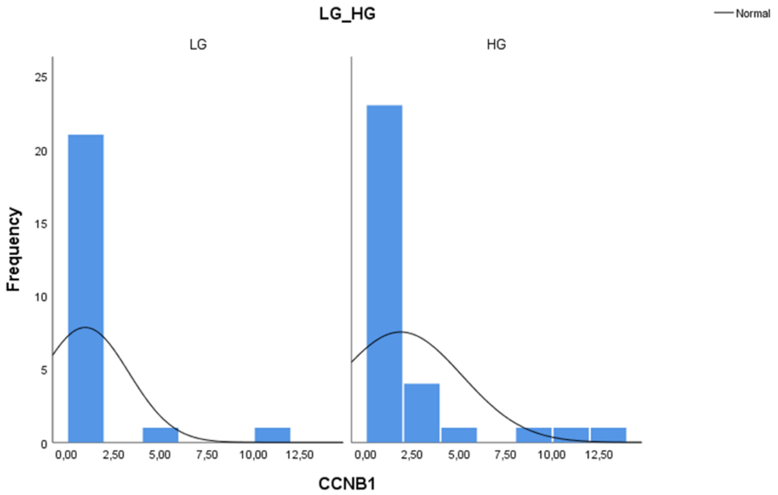

c.

Tests of Normality

	pTa_T1_T2	Kolmogorov-Smirnov[a] Statistic	df	Sig.	Shapiro-Wilk Statistic	df	Sig.
CCNB1	pTa	,330	36	,000	,489	36	,000
	pT1	,268	6	,200*	,915	6	,467
	pT2	,259	12	,025	,779	12	,005

*. This is a lower bound of the true significance.

a. Lilliefors Significance Correction

d.

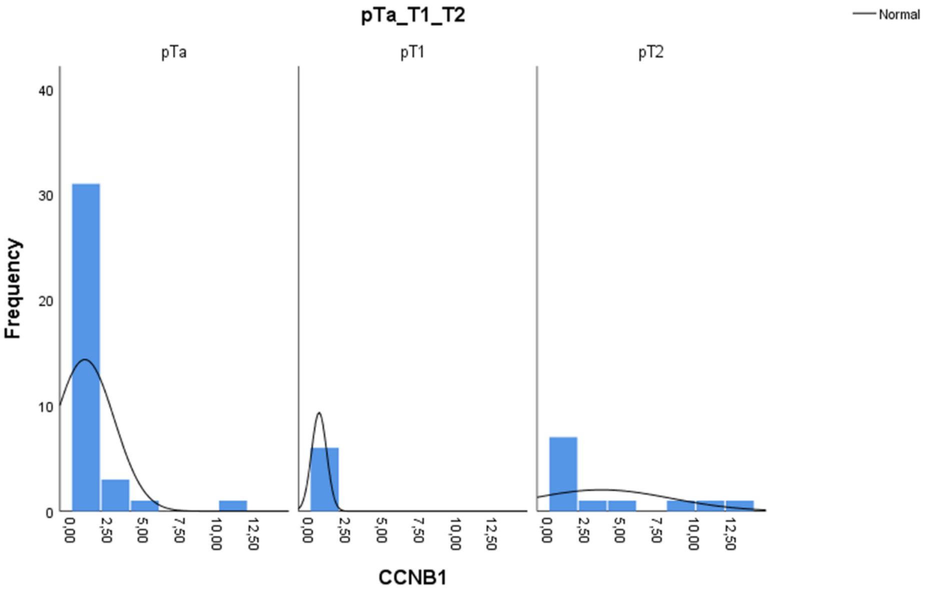

Supplementary Figure S4. Distribution and normality analysis of CCNB1 gene expression across LG, HG, and tumor staging groups (pTa, pT1, pT2). **a**. Distribution of fold changes in CCNB1 gene expression, **b.** Normality test for CCNB1 data in LG and HG groups, **c.** Distribution of CCNB1 gene expression fold-change in LG and HG groups, **d.** Normality test

for CCNB1 Data in the pTa, pT1, and pT2 groups **e.** Distribution of CCNB1 gene expression fold-change in the pTa, pT1, and pT2 groups.
